# Impacts of COVID-19 on Maternal and Newborn Healthcare Delivery in Central, Eastern and Southeastern Europe: A Scoping Review and Proposal for a Resilience Framework

**DOI:** 10.3390/medicina62101900

**Published:** 2026-10-01

**Authors:** Paula Trif, Radu Galiș, Diana Mudura, Boris W. Kramer, Liana Ștefan, Dana Carmen Zaha, Romina Viorela Murvai, Ioana Ștefăroi, Jan Mazela, Claudia Maria Jurca

**Affiliations:** 1 Doctoral School of Biological and Biomedical Sciences, University of Oradea, 410087 Oradea, Romania; 2 Department of Preclinical Disciplines, Faculty of Medicine and Pharmacy, University of Oradea, 410087 Oradea, Romania; 3 Department of Neonatology, Emergency County Hospital Bihor, 410169 Oradea, Romania; 4 Department of Medical Sciences, Faculty of Medicine and Pharmacy, University of Oradea, 410087 Oradea, Romania; 5 Department of Neonatology, Poznan University of Medical Sciences, 61-701 Poznan, Poland; 6 Department of Surgical Disciplines, Faculty of Medicine and Pharmacy, University of Oradea, 410087 Oradea, Romania; 7 Calla—Infertility Diagnostic and Treatment Center, 410103 Oradea, Romania; 8 Regional Center of Medical Genetics Bihor, County Emergency Clinical Hospital Oradea (Part of ERN ITHACA), 410169 Oradea, Romania

**Keywords:** COVID-19, healthcare resilience, health system preparedness, neonatal care, maternal health services

## Abstract

*Background and Objectives:* The COVID-19 pandemic profoundly disrupted maternal and newborn healthcare worldwide, necessitating rapid reorganization of healthcare services while maintaining essential care. Evidence from Central, Eastern and Southeastern Europe (CESEE) remains fragmented despite the region’s heterogeneous healthcare systems and variable pandemic responses. This scoping review synthesized the available evidence on maternal and newborn healthcare delivery during the COVID-19 pandemic and proposes a Maternal and Newborn Healthcare Resilience Framework (MNRF) to integrate the findings across key domains of healthcare resilience. *Materials and Methods:* PubMed/MEDLINE, Scopus, and Web of Science Core Collection were searched, with Google Scholar used as a supplementary search source. Eligible publications were published between 1 January 2020 and 31 May 2026. Evidence was synthesized across four predefined MNRF domains: access and continuity of care, healthcare organization and system capacity, quality and family-centered care, and maternal, perinatal, and neonatal outcomes. *Results:* Twelve publications met the inclusion criteria; several represented analyses derived from shared or partially overlapping datasets, particularly the multinational IMAgiNE EURO project. Across CESEE countries with available evidence, the pandemic disrupted access to antenatal services, accelerated the implementation of telemedicine, and prompted extensive reorganization of maternity and neonatal care. Family-centered practices, including birth companionship, skin-to-skin contact, breastfeeding support, and shared decision-making, were consistently affected. Maternal, perinatal, and neonatal outcomes showed no consistent regional pattern of deterioration in the included evidence; however, data were limited and heterogeneous, and localized increases in stillbirths and changes in neonatal healthcare utilization were reported. *Conclusions:* The COVID-19 pandemic exposed important vulnerabilities and prompted substantial adaptations in maternal and newborn healthcare delivery in the represented CESEE settings. The MNRF provides a structured, hypothesis-generating conceptual model for organizing these experiences and identifying priorities for preparedness and future research.

## 1. Introduction

According to the World Health Organization (WHO), health system resilience refers to the capacity of health systems to prepare for, respond to, and recover from crises while maintaining the delivery of essential health services [1,2,3]. Maternal and newborn healthcare constitutes one of the most essential and time-sensitive components of healthcare delivery. Unlike many medical services that can be postponed or reorganized during periods of healthcare disruption, pregnancy, childbirth, and neonatal care require continuous access to skilled professionals, specialized facilities, appropriate supplies and medications, and coordinated multidisciplinary care. Ensuring the continuity, accessibility, quality, and equity of maternal and newborn healthcare is essential for lowering maternal and neonatal morbidity and mortality, which is a key aspect of resilient healthcare systems. Maternal and newborn healthcare therefore represents a particularly sensitive indicator of health system resilience, as interruptions across the continuum of care may rapidly translate into adverse maternal and neonatal outcomes [4].

Public health emergencies exert considerable pressure on healthcare systems by increasing demand for healthcare resources, disrupting routine clinical pathways, reducing workforce capacity, and limiting access to essential services and supplies. Although infectious outbreaks primarily threaten population health through direct disease transmission, their indirect effects on healthcare delivery may be equally important [5]. Experiences from previous humanitarian crises and infectious disease outbreaks, including the Ebola epidemic, demonstrated that disruptions to routine maternal and newborn services frequently contributed more to maternal and neonatal morbidity and mortality than the infectious disease itself [6]. Consequently, preserving essential maternal and newborn healthcare has become a central component of international preparedness strategies for future public health emergencies [7,8,9].

The COVID-19 pandemic represented the largest global stress test experienced by modern healthcare systems. Hospitals were required to rapidly redistribute resources, implement infection-prevention measures, reorganize clinical pathways, and manage unprecedented workforce shortages while simultaneously maintaining essential healthcare services. Maternal and neonatal care services were no exception. Across the world, maternity units and neonatal intensive care units implemented substantial organizational changes to ensure safe care delivery while minimizing viral transmission. Across the maternal–newborn continuum of care, numerous adaptations were rapidly introduced to balance infection control with the continuity of essential healthcare delivery. Many countries reduced face-to-face antenatal consultations, expanded telemedicine services, modified referral pathways, restricted the presence of birth companions, reorganized maternity wards and neonatal intensive care units, implemented visitor limitations, and revised recommendations regarding breastfeeding, rooming-in, and postnatal discharge [6,10,11]. While several of these measures successfully reduced opportunities for viral transmission and accelerated innovation in healthcare delivery, others unintentionally affected access to care, communication, emotional support, bonding, and the overall experience of pregnancy and childbirth.

Among the most important challenges identified during the pandemic was the preservation of family-centered maternal and newborn care, an internationally recognized approach that promotes parental involvement, early mother–infant interaction, breastfeeding support, and shared decision-making. Infection control measures frequently resulted in restrictions on birth companionship, reduced parental presence within neonatal intensive care units, limitations to skin-to-skin contact, changes in rooming-in practices, and decreased opportunities for breastfeeding support [12].

Considerable attention has also been directed toward understanding whether these healthcare adaptations influenced maternal, perinatal, and neonatal outcomes. While reductions in preterm birth were described in several high-income countries, other studies documented stable or increased rates of adverse perinatal outcomes [6,13,14,15,16,17]. Similarly, systematic reviews and multinational analyses have demonstrated substantial heterogeneity, suggesting that differences in healthcare organization, socioeconomic context, public health policies, and baseline healthcare capacity may explain much of the observed variation rather than the direct biological effects of COVID-19 infection alone [18,19,20]. These observations reinforce the need to evaluate maternal and newborn healthcare from a broader health system perspective rather than focusing exclusively on infection-related outcomes.

European countries exhibit considerable heterogeneity in healthcare organization, healthcare expenditure, workforce capacity, digital health implementation, and socioeconomic conditions. Many countries share similar historical and political backgrounds, but important differences exist in the organization of maternity and neonatal services, urban–rural healthcare access, and resource availability. These contextual factors may have influenced how healthcare systems responded to the COVID-19 pandemic and how maternal and newborn healthcare delivery was maintained during the crisis [19,21].

Although numerous reviews have examined maternal COVID-19 infection, pregnancy outcomes, and neonatal infection, evidence on the broader impact of the pandemic on maternal and newborn healthcare delivery in CESEE remains insufficiently synthesized. Comparative evidence across CESEE healthcare systems is limited, maternal and neonatal outcomes have not been reported using standardized measures, and dimensions such as family experience and quality of care have received less attention than infection-related outcomes. These gaps make it difficult to determine which aspects of maternal and newborn healthcare were most vulnerable to disruption and which system-level adaptations supported continuity and resilience during the pandemic. Therefore, this scoping review aims to map and synthesize the available evidence on maternal and newborn healthcare delivery during the COVID-19 pandemic in CESEE, encompassing healthcare access and continuity, healthcare organization and system capacity, quality and family-centered care, and maternal, perinatal, and neonatal outcomes. To integrate these interconnected dimensions, we propose the Maternal and Newborn Healthcare Resilience Framework (MNRF) as a structured approach for interpreting healthcare-system responses and identifying lessons that may inform policy, future research, and preparedness for public health emergencies.

## 2. Materials and Methods

### 2.1. Study Design

For the purposes of this review, Central, Eastern and Southeastern Europe (CESEE) comprised Albania, Bosnia and Herzegovina, Bulgaria, Croatia, Czech Republic, Estonia, Hungary, Latvia, Lithuania, Moldova, Montenegro, North Macedonia, Poland, Romania, Serbia, Slovakia, and Slovenia. This predefined country list constituted the geographical eligibility criterion applied throughout study selection. The methodological framework originally proposed by Arksey and O’ Malley [22] and further refined by Levac et al. was followed [23]. The review process included the following stages: (1) identification of the research question, (2) identification of relevant studies, (3) study selection, (4) data charting, and (5) collation, synthesis, and reporting of the results. PRISMA Extension for Scoping Reviews (PRISMA-ScR) standards were followed [24].

No a priori protocol for this scoping review was registered or published.

### 2.2. Research Question and Conceptual Framework

The review question and eligibility criteria were developed using the Population–Concept–Context (PCC) framework for scoping reviews [25].

The review sought to answer the following question: what evidence exists regarding maternal and newborn healthcare delivery during the COVID-19 pandemic in CESEE, and what lessons can be drawn to strengthen healthcare resilience during future public health emergencies?

The PCC framework guiding the review is presented in Table 1.

The four MNRF domains were established beforehand during the development of the review question and PCC framework, prior to selecting studies and synthesizing evidence. These domains were specifically created by the review authors for this review and were not borrowed from an existing resilience framework, derived inductively from the included evidence, or developed through external expert or stakeholder consensus. They were based on the dimensions of maternal and newborn healthcare delivery that the review was designed to examine. The MNRF was therefore used as a structured, hypothesis-generating conceptual model for organizing and interpreting heterogeneous evidence rather than as a validated instrument for measuring healthcare-system resilience. No formal external validation, stakeholder consensus process, or prospective testing of the MNRF was undertaken. The framework organizes the available evidence into four interconnected domains representing essential components of resilient maternal and newborn healthcare systems:

1. Access and continuity of care;
2. Healthcare organization and system capacity;
3. Quality and family-centered care;
4. Maternal, perinatal, and neonatal outcomes.

For evidence mapping, the four domains were operationally defined as follows: (1) access and continuity of care encompassed availability, accessibility, attendance, and continuity of maternal and newborn healthcare services, including antenatal care, screening, and access to essential services; (2) healthcare organization and system capacity encompassed changes in service organization and delivery, healthcare pathways, staffing or resource allocation, infection-control arrangements, telemedicine, and other adaptations implemented to maintain service provision; (3) quality and family-centered care encompassed the experience and quality of care, including communication, respectful care, companionship, shared decision-making, mother–infant contact, skin-to-skin care, rooming-in, and breastfeeding support; and (4) maternal, perinatal, and neonatal outcomes encompassed reported maternal, pregnancy, birth, perinatal, and neonatal outcomes and patterns of healthcare utilization relevant to the pandemic period.

Within this review, healthcare-system adaptation refers to organizational or service-delivery changes implemented in response to pandemic-related pressures, whereas resilience is understood more broadly as the capacity to absorb disruption, adapt, and maintain essential healthcare functions during a crisis. Accordingly, the presence of adaptive measures was not considered, by itself, evidence that a healthcare system was resilient. The MNRF was used to organize evidence relevant to different dimensions of resilience rather than to classify individual healthcare systems as resilient or non-resilient.

**Figure 1:**
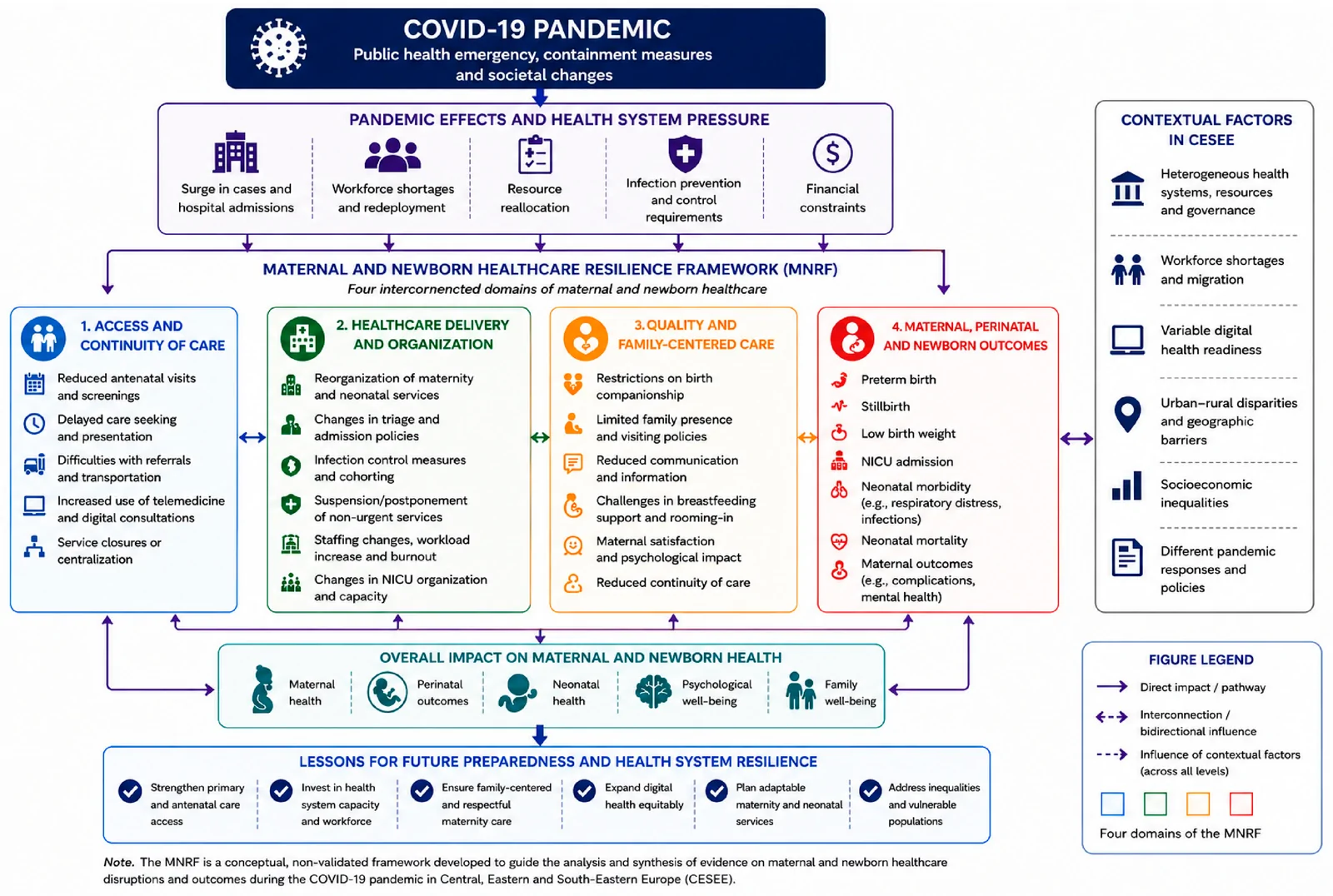
Conceptual framework illustrating the pathways through which the COVID-19 pandemic influenced maternal and newborn healthcare delivery and associated outcomes in CESEE.

Each included publication was assigned to one or more of the predefined MNRF domains according to the operational definitions above (Figure 1).

### 2.3. Search Strategy

A comprehensive literature search was conducted in three bibliographic databases—PubMed/MEDLINE, Scopus, and Web of Science Core Collection—with Google Scholar used as a supplementary search source, for publications meeting the predefined eligibility period of 1 January 2020 to 31 May 2026. Search strategies combined controlled vocabulary, where applicable, with free-text terms related to COVID-19, maternal and newborn healthcare, healthcare delivery, and the predefined CESEE countries. Search syntax was adapted to the indexing and search functions of each source. The available source-specific search strategies are provided in the Supplementary Materials. Google Scholar was used as a supplementary search source to increase the retrieval of potentially relevant national and regional literature that may be incompletely represented in major bibliographic databases. Because Google Scholar results are relevance-ranked and may vary over time, screening was limited to the first 200 results returned by each predefined search. The same eligibility criteria used for the bibliographic database searches were applied during Google Scholar screening. The gray literature was not systematically searched. The complete search strategies for all databases are provided in the Supplementary Materials.

### 2.4. Eligibility Criteria

Studies were considered eligible if they fulfilled all of the following inclusion criteria.

Inclusion criteria

Studies were included if they

• Were published between 1 January 2020 and 31 May 2026;
• Were published in English;
• Reported original research;
• Included data from one or more countries from CESEE;
• Evaluated one or more aspects of maternal or newborn healthcare delivery during the COVID-19 pandemic;
• Reported maternal, perinatal, or neonatal outcomes relevant to understanding the consequences of healthcare-system adaptations during the pandemic.

Eligible study designs included prospective and retrospective cohort studies, case–control studies, cross-sectional surveys, registry-based investigations, qualitative studies, and mixed-methods studies.

Exclusion criteria

Studies were excluded if they

• Did not include countries in the predefined geographical region;
• Did not report original data;
• Focused exclusively on maternal COVID-19 infection, virology, laboratory findings, pharmacological treatment, or vaccine effectiveness without evaluating healthcare delivery or reporting relevant maternal or neonatal outcomes;
• Were studies without full-text access;
• Were published in languages other than English.

Studies directly evaluating maternal and newborn healthcare delivery constituted the primary evidence base. Observational studies reporting maternal, perinatal, or neonatal outcomes were also retained when these outcomes were relevant to the review question. Such findings were used to describe patterns occurring during the pandemic period and were not interpreted as demonstrating effects of healthcare-system adaptations or disruptions.

### 2.5. Study Selection

**Figure 2:**
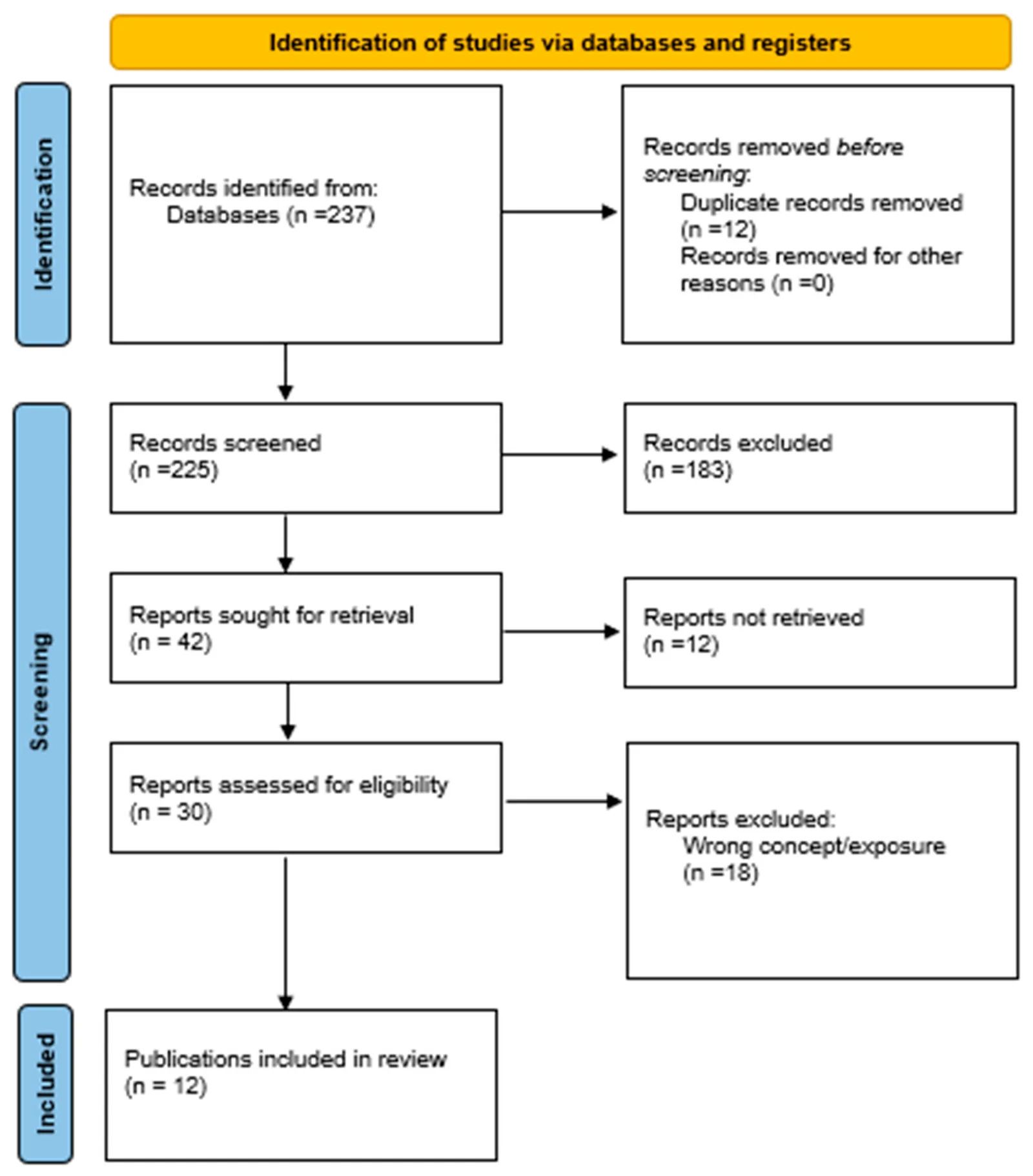
PRISMA flow diagram.

Two reviewers (R.V.M and R.G.) independently screened titles and abstracts according to the predefined eligibility criteria. Potentially relevant articles underwent full-text assessment. Disagreements regarding study eligibility were resolved through discussion until consensus was reached; when consensus could not be reached, a third reviewer was consulted. The study selection process is presented using the PRISMA-ScR flow diagram (Figure 2) [24].

### 2.6. Data Charting

A standardized data extraction form was developed before study selection. For each included study, the following information was extracted:

• First author;
• Year of publication;
• Country;
• Study design;
• Study population;
• Sample size;
• Study objectives;
• Healthcare adaptations or domains evaluated;
• Principal findings.

Each study was subsequently mapped to one or more domains of the MNRF by two reviewers (R.V.M. and R.G.) according to the operational definitions in Section 2.2. Publications evaluating multiple aspects of healthcare delivery were classified across more than one domain when appropriate. Disagreements in domain assignment were resolved through discussion and consensus, with consultation of a third reviewer when required.

The study period and, where reported, the pandemic phase or relevant policy context (e.g., strict lockdown) were also considered during data charting and interpretation. Temporal distinctions reported by the original publications were preserved in the synthesis when available; however, studies were not retrospectively assigned to standardized pandemic phases because observation periods and policy contexts differed across countries and publications.

### 2.7. Quality Appraisal

Methodological quality was independently assessed by two reviewers (R.V.M. and R.G.) using the Joanna Briggs Institute (JBI) critical appraisal checklist appropriate to each study design. Individual checklist items were evaluated as Yes, No, Unclear, or Not applicable. Disagreements between the two reviewers were resolved through discussion and consensus; when consensus could not be reached, a third reviewer was consulted. The appraisal was undertaken to characterize the methodological strengths and limitations of the available evidence and to contextualize its interpretation. Studies were not excluded or formally weighted according to appraisal findings. Detailed item-level assessments are provided in Supplementary Table S1.

## 3. Results

### 3.1. Study Selection

The electronic database search identified 237 records. After the removal of duplicates, 225 titles and abstracts were screened for eligibility. Full-text assessment was performed for 30 articles, of which 12 met the predefined inclusion criteria and were included in the final synthesis.

### 3.2. Characteristics of Included Studies

This scoping review included a total of 12 eligible publications published from 2022 to 2025 (Table 2), representing evidence from several distinct studies and datasets across CESEE. The geographical distribution of the evidence was uneven. Country-specific or subregional eligible publications were concentrated in seven of the 17 predefined CESEE countries, whereas several other eligible countries were represented only, if at all, within multinational analyses (Supplementary Table S2).

Four publications were derived from the multinational IMAgiNE EURO project and included multicountry, subregional, or country-specific analyses of the same overarching survey dataset. In addition, the two Romanian population-based publications included partially overlapping data from Bihor County. These related publications were retained because they addressed different geographical subsets, outcomes, or analytical questions, but their non-independence was considered when synthesizing the evidence.

Using the MNRF, each included study was mapped to one or more of four interconnected domains: (1) access and continuity of care, (2) healthcare organization and system capacity, (3) quality and family-centered care, and (4) maternal, perinatal, and neonatal outcomes (Table 2). Several studies contributed to multiple domains, reflecting the complex interplay between healthcare organization, quality of care, and clinical outcomes during the COVID-19 pandemic.

Overall, studies evaluating healthcare organization and quality of maternity care predominated, particularly through the multinational IMAgiNE EURO initiative, whereas evidence regarding long-term neonatal outcomes, healthcare workforce resilience, and post-pandemic recovery strategies remained comparatively limited.

The included evidence was methodologically heterogeneous and comprised cross-sectional surveys of maternal care experiences, administrative and registry-based analyses of healthcare utilization and population-level outcomes, observational cohort studies, and prenatal screening data. These evidence sources addressed distinct populations and research questions and were interpreted according to their respective study design, population, data source, and outcome type rather than as methodologically equivalent evidence. Studies specifically investigating clinical outcomes associated with maternal SARS-CoV-2 infection were outside the scope of the review; where SARS-CoV-2-positive populations were included, eligibility was based on the evaluation of healthcare delivery or perinatal management practices rather than infection-related clinical outcomes. The subsequent MNRF domain-based synthesis therefore provides a conceptual organization of heterogeneous evidence and does not imply pooling or direct comparability across study designs.

### 3.3. Impact of the COVID-19 Pandemic on Maternal and Newborn Healthcare Delivery

The findings were synthesized according to the four domains of the MNRF. Of the 12 included publications, six contributed evidence on access and continuity of care, eight on healthcare organization and system capacity, five on quality and family-centered care, and four on maternal, perinatal, and neonatal outcomes. Several studies contributed to more than one domain (Table 2). Because some publications originated from shared or partially overlapping datasets, these counts describe the distribution of publications across MNRF domains rather than the number of independent evidence sources.

#### 3.3.1. Access and Continuity of Maternal Healthcare During the COVID-19 Pandemic

Six publications evaluated the impact of the COVID-19 pandemic on access and continuity of maternal healthcare, including five cross-sectional surveys and one nationwide screening program analysis.

The COVID-19 pandemic substantially affected access to maternal healthcare across the represented CESEE settings. Although the extent of disruption varied between countries, the included studies consistently reported interruptions to routine antenatal care, postponement or cancelation of scheduled visits, reduced availability of face-to-face consultations, restrictions on partner attendance, and increased reliance on remote healthcare delivery [21].

Evidence from the multinational IMAgiNE EURO studies demonstrated that disruptions to antenatal care were common across Europe, with healthcare systems from countries such as Romania, Croatia, and Serbia showing worse indicators when the quality of maternal and newborn care (QMNC) was evaluated. In the domain of reorganizational changes due to COVID-19, 55.6% of the Romanian participants reported a reduction in the quality of care, compared with 85% in Slovenia, 60% in Serbia, and 70% in Croatia. In Romania and Serbia, over 55% of participants reported difficulties attending routine antenatal visits, whereas in Croatia and Slovenia, only about 40% did so. In addition, a substantial proportion of women reported inadequate communication with healthcare professionals to address COVID-19-related stress, with higher gaps in Serbia and Bosnia–Herzegovina. An inadequate number of healthcare personnel ranged from 32.1% in Romania to 53.8% in Bosnia–Herzegovina [26,27].

After adjustment for potential confounders, multivariable analyses revealed significant variation in the QMNC index across countries, with healthcare systems in Croatia, Romania, and Serbia having significantly lower QMNC indexes (270, 275, and 205, on a scale ranging from 0 to 400, with higher scores indicating better reported quality of maternal and newborn care). These findings support the hypothesis that QMNC may vary according to geographical or geopolitical region [21,26,27]. Moreover, data evaluating the Latvian healthcare system demonstrates that the QMNC index for reorganizational changes due to COVID-19 improved in 2021 compared to 2020 (*p* < 0.001) [28].

National studies further illustrated the heterogeneity of healthcare adaptations. In Poland, Kalita-Kurzyńska et al. reported that almost all surveyed women (95.6%) experienced changes in maternity care during the first pandemic wave. The most frequently reported modifications included restrictions on partner attendance during antenatal consultations and childbirth (87.7%), particularly in private healthcare services (93%). Cancelation of scheduled appointments was reported by 36% of National Health System users and 53% of those with private healthcare insurance. Problems accessing laboratory tests, fewer prenatal consultations, and reduced consultation duration or fetal ultrasound were also experienced. Despite these changes, telemedicine enabled many women to maintain contact with healthcare professionals when face-to-face consultations were limited, demonstrating its value as an emergency strategy rather than a complete substitute for routine maternity care. More than half of the participants (55.2%) reported using teleconsultations or e-consultations during the COVID-19 pandemic, with no differences regarding the maternity care financing model used [29].

Using nationwide data from the Polish Prenatal Screening Program, Czuba et al. also demonstrated disruptions in preventive maternity services. Participation in prenatal screening in 2020 was reduced by 12.4% compared with 2019, particularly among women under 35 years of age. The authors also reported fewer invasive diagnostic procedures and first-trimester examinations [30]. These findings suggest that the indirect consequences of the pandemic extended beyond routine obstetric consultations and could have influenced access to specialized prenatal diagnostic services.

Overall, the available evidence indicates that maintaining uninterrupted access to antenatal and preventive maternity services represented one of the greatest challenges during the COVID-19 pandemic across CESEE nations with available evidence, despite the rapid implementation of adaptive strategies such as telemedicine and modified care pathways.

#### 3.3.2. Healthcare Organization and System Capacity During the COVID-19 Pandemic

Healthcare organization and system capacity was the most extensively investigated domain, with evidence derived from eight included studies. The available literature comprised multinational surveys, national registry analyses and cross-sectional studies, providing complementary perspectives on how maternity and neonatal services adapted during the pandemic.

Healthcare systems were required to balance infection-prevention measures with the maintenance of essential maternal and newborn services, resulting in substantial modifications to hospital organization, patient flow, visitor policies, and care models. Although the specific strategies varied across countries, several common organizational adaptations emerged.

Evidence from the multinational IMAgiNE EURO project consistently demonstrated that infection-control measures substantially influenced the organization of maternity services. Many hospitals implemented restrictions on support persons during labor and childbirth, modified admission procedures, and introduced changes in postpartum care pathways [21].

Regional analyses from Slovenia, Croatia, Serbia, and Bosnia and Herzegovina reported inadequate ward organization (range: 28.2% in Slovenia to 73.0% in Bosnia–Herzegovina) and room reorganization (range: 42.3% in Slovenia to 74.2% in Bosnia and Herzegovina), with overcrowded rooms ranging from 9.7% in Romania to 21.4% in Serbia [26,27]. Although hospitals adapted their organization according to local epidemiological conditions and national public health policies, restrictions on partner visiting remained widespread. Inadequate partner visiting hours were reported by the majority of women across all countries, with rates ranging from 80.7% in Slovenia to 90.4% in Serbia.

The availability of physical and human resources was also assessed in the IMAgiNE EURO study. Overall, participating women reported several deficiencies in care provision, including delays in receiving timely care from healthcare professionals upon hospital arrival (lowest in Slovenia, 12.3%, and highest in Serbia, 34.4%), inadequate information on maternal danger signs (over 40% in all CESEE countries), and inadequate information on newborn danger signs (over 45% across participating CESEE) [26,27].

Hospital-level adaptations were also evident in Latvia. Between 2020 and 2021, significant improvements were observed in the provision of care and the availability of physical and human resources. Accordingly, both the overall QMNC index and the indexes for each subdomain increased significantly in 2021 compared with 2020 (*p* < 0.001) [28].

In addition to organizational changes, the multinational IMAgiNE EURO study evaluated infection-prevention practices, including healthcare professionals’ use of personal protective equipment. A proportion of participating women reported that healthcare workers did not always wear appropriate protective equipment during care. The percentage varied across countries, from 16.5% for Romania to 39.2% in Bosnia–Herzegovina [21].

National healthcare utilization data provided further evidence of organizational restructuring.

In Croatia, substantial reductions in obstetric and gynecological hospital admissions during the pandemic were observed. Analyses of Diagnosis-Related Group data demonstrated a marked 6.64% reduction in the surgical treatment of conditions related to pregnancy and childbirth, and an almost 20% reduction in the number of patients treated at the tertiary healthcare level compared to the previous year [33]. While part of this reduction likely reflected healthcare-system reorganization and infection-control measures, changes in healthcare-seeking behavior may also have contributed to decreased utilization of hospital services.

The reduction in prenatal screening activity reported in Poland’s healthcare system also reflected the reorganization of maternity services during the pandemic, with healthcare resources redirected toward pandemic response and non-urgent diagnostic services temporarily reduced [30]. Hospitals restructured antenatal care pathways by expanding telemedicine, modifying consultation schedules, and introducing infection-control measures, including restrictions on birth companions and visitor access. These changes enabled the continuation of essential maternity services while reducing opportunities for face-to-face interactions [29]. These changes could have impacted neonatal hospitalizations during the pandemic. A national registry data from Poland demonstrated a substantial increase in neonatal hospitalizations during the pandemic, particularly in 2021 (77.7‰ vs. 50.3–51.9‰ in 2017–2019), despite declining birth rates (mean birth rate 331,511 in 2021 vs. a mean birth rate of 388,371 for 2017–2019). The authors suggested that healthcare reorganization, heightened clinical vigilance, and changes in maternal health may have contributed to increased demand for neonatal hospital care [32].

Collectively, these findings indicate substantial reorganization of maternity services in the represented settings; however, the degree and form of adaptation varied across countries and should not, by itself, be interpreted as evidence of healthcare-system resilience.

#### 3.3.3. Quality and Family-Centered Care During the COVID-19 Pandemic

Five studies examined the quality of maternal and newborn care and family-centered practices during the COVID-19 pandemic. Most were cross-sectional surveys exploring women’s experiences of childbirth, supplemented by one prospective observational study evaluating the feasibility of maintaining evidence-based newborn care practices among mothers with SARS-CoV-2 infection.

Beyond maintaining access to maternity services, preserving the quality of maternal and newborn care emerged as a major challenge during the COVID-19 pandemic. While healthcare systems generally succeeded in maintaining essential clinical services, several components of family-centered maternity care were compromised, particularly during the early phases of the pandemic.

The multinational IMAgiNE EURO studies identified considerable variability in the quality of maternal and newborn care among participating countries [21].

The majority of survey participants reported being unable to move freely during labor (45.2% in Romania, 51.7% in Slovenia, 52.1% in Bosnia–Herzegovina, and 68.0% in Serbia). Pain relief during labor for vaginal birth was missing for 21.2% of respondents in Slovenia, 43.3% of respondents in Romania, and over 65% in Bosnia–Herzegovina. A lack of attention when needed was also reported, with rates ranging from 27% to 63.3% depending on the country and type of birth [26,27].

Otelea et al. reported that women giving birth in Romania during the pandemic frequently experienced reduced opportunities for skin-to-skin contact (68.2%), delayed initiation of breastfeeding (68.5%), and inconsistent rooming-in practices (48.1%), with even worse scores among women who underwent cesarean delivery [27]. Reports of no skin-to-skin contact after birth were also recorded in Bosnia–Herzegovina, 45.5%, whereas in Slovenia the percentage was much lower, at only 14.1%. Similarly, the absence of early breastfeeding varied from 13.0% in Slovenia to 71.8% in Serbia, while the lack of exclusive breastfeeding at discharge ranged from 36.0% in Slovenia to 45.8% in Croatia. In Romania, values reached over 50%.

Similarly, Latvian women participating in the IMAgiNE EURO project reported limitations in pain control during birth, breastfeeding support, lack of attention from healthcare personnel when needed, and variable adherence to WHO quality standards during childbirth and the immediate postpartum period. Importantly, the Latvian study also demonstrated progressive improvement in the above-mentioned care indicators between successive phases of the pandemic [28].

Disrespectful care was frequently reported, with the proportion of women stating that they were not treated with dignity and respect ranging from 21.5% in Slovenia to 62.8% in Serbia. Reports of physical, verbal, or emotional abuse also varied across countries, from 11.0% in Slovenia to 30.5% in Bosnia and Herzegovina. Substantial between-country differences were also observed regarding lack of privacy, which ranged from 17.7% in Slovenia to 69.2% in Serbia [21,26,27].

Family involvement represented one of the domains most consistently affected across the region. While these restrictions were introduced for infection-control purposes, several studies reported that women perceived reduced emotional support during labor and childbirth, particularly in Bosnia–Herzegovina (60.3%) and Serbia (61.3%) [26].

The limited companionship indicator showed the greatest improvement among the assessed quality measures in Latvia when 2020 was compared to 2021 (falling from 43.1% to 30.7%) [28].

Communication and women’s involvement in decision-making also varied considerably across countries. The proportion of women reporting a lack of clear or effective communication with healthcare providers ranged from 31.1% in Slovenia to 58.8% in Serbia, while the proportion reporting not being involved in decisions regarding their care ranged from 31.1% in Slovenia to 78.3% in Serbia [26].

Survey data suggested that effective communication was closely associated with women’s satisfaction with maternity care despite the challenges imposed by the pandemic.

Despite these challenges, several studies have documented successful preservation of evidence-based maternity and newborn care. Prospective observational data from Poland demonstrated that, even among mothers with confirmed SARS-CoV-2 infection, hospitals were able to maintain high rates of rooming-in (83.3%), early skin-to-skin contact (83.3%), and breastfeeding (84.5%) when appropriate infection-control measures were implemented [31].

Together, these studies suggest that family-centered care represented one of the most vulnerable dimensions of maternity services during the pandemic, despite increasing evidence supporting the safe continuation of many evidence-based newborn care practices.

#### 3.3.4. Maternal, Perinatal and Neonatal Outcomes During the COVID-19 Pandemic

Four publications reported maternal, perinatal, or neonatal outcomes relevant to healthcare-system adaptations during the COVID-19 pandemic. These included population-based cohort studies, national registry analyses, and retrospective observational studies evaluating both regional trends and healthcare utilization.

Although the primary focus of this review was healthcare delivery, several included publications also reported maternal, perinatal, and neonatal outcomes occurring during the pandemic period. These findings were included to describe concurrent outcome patterns and were not interpreted as demonstrating effects of healthcare-system adaptations or disruptions.

Population-based studies from Romania provided valuable insights into regional trends. Population-based studies from Romania provided valuable insights into regional trends. Galis et al. observed that, while preterm birth rates decreased slightly during the pandemic (RR = 0.88), stillbirths increased compared with the pre-pandemic period (RR = 1.53 (95% CI 1.05–2.23)). The most frequently identified cause of stillbirth was asphyxia due to placental pathology (67.6%), and no differences in maternal characteristics of stillborn infants were found when the pandemic was compared to the pre-pandemic period [15]. Trif et al. analyzed a large cohort of 52,021 newborns, comparing preterm birth and stillbirth rates across pre-pandemic, pandemic, and post-pandemic periods in two counties of Romania. Trif et al. analyzed a large cohort of 52,021 newborns, comparing preterm birth and stillbirth rates across pre-pandemic, pandemic, and post-pandemic periods in two counties of Romania. Stillbirth increased transiently during the strict lockdown period (*p* = 0.05), whereas preterm birth remained stable across the six-year study period (*p* = 0.13) [16].

Dagelić et al. found no significant differences in miscarriage (*p* = 0.267), stillbirth (*p* = 0.309), or early neonatal mortality (*p* = 0.605) between the pre-pandemic and pandemic periods in Croatia [34].

Nationwide data from Poland further showed that neonatal hospitalization patterns changed considerably during the pandemic. Although no significant increase in prematurity or neonatal mortality was observed, neonatal hospitalization rates increased during the pandemic. Hospitalizations involving newborns affected by maternal renal and urinary tract diseases (*p* < 0.001), maternal complications of pregnancy (*p* < 0.001), complications of the placenta, cord, and membranes (*p* < 0.001), and gestational diabetes (*p* < 0.001) were significantly more frequent during the pandemic than in the pre-pandemic period [32]. These findings suggest changes in neonatal healthcare utilization and case mix rather than a generalized deterioration in neonatal outcomes.

Overall, the available evidence did not consistently demonstrate widespread deterioration in the examined maternal, perinatal, and neonatal outcomes. However, outcome evidence was limited and heterogeneous, and adverse changes, including increased stillbirths, were reported in specific settings and periods.

## 4. Discussion

This scoping review synthesizes evidence on maternal and newborn healthcare delivery during the COVID-19 pandemic across CESEE using a structured conceptual model. The evidence base comprised 12 eligible publications, although several originated from shared or partially overlapping datasets, particularly the multinational IMAgiNE EURO project. Overall, the available evidence indicated disruptions in access and continuity of care, substantial organizational adaptations, and adverse effects on several components of quality and family-centered care. Maternal, perinatal, and neonatal outcomes showed no consistent pattern of deterioration in the represented settings; however, evidence in this domain was comparatively limited and heterogeneous, and adverse changes were reported in specific settings.

Throughout the pandemic, the World Health Organization (WHO) consistently emphasized that essential maternal and newborn healthcare services should be maintained, even for women with suspected or confirmed COVID-19 infection. WHO recommendations advocated for the continuation of routine antenatal care, respectful maternity care, effective communication, companionship during labor, early skin-to-skin contact, rooming-in, and breastfeeding whenever clinically appropriate, highlighting that infection-prevention measures should not unnecessarily compromise evidence-based maternity and newborn practices [35,36]. One of the principal findings of this review was that maintaining uninterrupted access to essential maternal healthcare represented a major challenge during the COVID-19 pandemic across CESEE. Disruptions were consistently reported in antenatal care attendance, prenatal screening programs, and continuity of routine maternity services, although the extent of these disruptions varied among healthcare systems [26,27,28,29,30]. Our findings are consistent with the broader international literature [10]. A large systematic review and meta-analysis by Townsend et al. included 56 studies and demonstrated that the COVID-19 pandemic resulted in a significant global reduction in scheduled antenatal visits, decreased uptake of prenatal screening, and widespread changes in maternity healthcare-seeking behavior, accompanied by a marked increase in remote consultations [37]. In our review, several studies described the replacement of face-to-face antenatal consultations with remote visits to ensure continuity of care while minimizing exposure to COVID-19. Subsequent systematic reviews have suggested that hybrid models combining in-person and virtual antenatal care can achieve maternal and neonatal outcomes comparable to traditional care. Nevertheless, current evidence also highlights persistent concerns regarding equitable access, patient experience, and the ability of remote care to fully replace clinical assessment, particularly among socially vulnerable populations or women with high-risk pregnancies [38,39,40].

The organizational changes identified across CESEE were not unique to the region. International evidence similarly documented fewer antenatal and unscheduled visits, increased remote care, and substantial changes in how maternity services were delivered during the pandemic. UK surveys reported service reductions that varied between and within regions [41],while Italian maternity services were reorganized by introducing designated referral pathways, separate care areas for infected and non-infected women, universal screening, and dedicated clinical protocols. In the reported Italian settings, these organizational changes were implemented while the examined maternal and neonatal outcomes remained stable, although observational evidence cannot establish that the adaptations themselves caused those outcomes [42,43].

These experiences illustrate potential components of preparedness and adaptation but should not be interpreted as demonstrating resilience solely because services were reorganized or remained operational. Future emergency preparedness should consider predefined referral networks, protected maternity staffing, scalable infection-control pathways, reliable communication between levels of care, and sufficient flexibility to preserve community-based and family-centered models whenever clinically safe [12].

Notably, international evidence indicates that pregnant women frequently delayed or avoided healthcare because of concerns regarding COVID-19 exposure, uncertainty surrounding hospital policies, and fear of infection. Consequently, preserving access during future public health emergencies requires not only maintaining healthcare capacity but also ensuring clear communication, public confidence, and timely dissemination of evidence-based recommendations [37].

One of the most important findings of this review is that maintaining access to maternity services did not necessarily guarantee the preservation of high-quality, family-centered care. Although most healthcare systems successfully continued essential obstetric and neonatal services throughout the pandemic, many women reported substantial changes in their childbirth experience [21,26,27,28]. WHO guidance emphasized respectful maternity care, effective communication, companionship during labor, early skin-to-skin contact, rooming-in, and breastfeeding whenever clinically appropriate [36,44]. External international evidence also documented an evolution in recommendations regarding breastfeeding and mother–infant separation as knowledge of SARS-CoV-2 transmission increased [45,46]. Importantly, organizational adaptation and continuity of essential services should not be considered synonymous with resilience. Deterioration in several aspects of family-centered care despite continued service provision indicates that healthcare systems may preserve operational capacity while compromising other dimensions of care. Within the MNRF, family-centered care therefore represents a distinct and complementary dimension relevant to understanding maternal and newborn healthcare resilience. Maternal, perinatal, and neonatal outcomes provide an important complementary perspective on the pandemic period, but the observational evidence identified in this review does not permit these outcomes to be used as direct measures of the effectiveness of healthcare-system adaptations. Studies from Romania and Croatia reported largely stable rates of several examined outcomes, including preterm birth, miscarriage, and neonatal mortality, although temporary increases in stillbirths were observed in specific Romanian settings [15,16,34,47].

Nationwide data from Poland indicated changes in neonatal hospitalization patterns without substantial deterioration in the examined neonatal outcomes [32]. Changes in healthcare utilization should be interpreted cautiously, as differences in hospitalization rates may reflect changes in case mix, referral pathways, admission thresholds, healthcare access, or ascertainment rather than changes in healthcare quality or system performance alone. These findings should be interpreted as changes observed during the pandemic period rather than as outcomes causally attributable to the healthcare-system adaptations or disruptions identified in the other MNRF domains. A landmark systematic review and meta-analysis by Chmielewska et al. demonstrated increases in stillbirths and maternal mortality predominantly in low- and middle-income countries, underscoring the importance of healthcare-system and socioeconomic context when interpreting heterogeneous pandemic-era outcome patterns [48]. Similar findings were reported in the Netherlands or Jordan, where no significant changes in preterm birth, stillbirth, neonatal mortality, or other major perinatal outcomes were observed [13,14]. Likewise, the Euro-Peristat collaboration reported only modest changes in preterm birth and stillbirth rates across 21 European countries, whereas Nepal experienced increased stillbirths and neonatal mortality following a marked reduction in institutional births during the national lockdown [6,19].

A major finding of this review is the marked geographical imbalance of the available evidence. Despite the broad predefined CESEE scope, country-specific or subregional evidence was concentrated in a relatively small number of countries, while several countries were not represented by any eligible publication outside multinational analyses. Consequently, the findings should not be interpreted as characterizing maternal and newborn healthcare systems across CESEE as a whole. Rather, they describe patterns observed in the represented settings and reveal an important regional evidence gap. This imbalance limits meaningful cross-country comparisons and highlights the need for coordinated multicountry research using standardized measures of healthcare delivery, family-centered care, and maternal and neonatal outcomes. The available evidence also provides limited insight into how disruptions and adaptations affected socially vulnerable, rural, or otherwise underserved populations, making equity an important priority for future research.

Taken together, the findings indicate that preparedness for future public health emergencies should address more than service availability and should also consider equitable access, evidence-based clinical practice, effective communication, and family-centered care. The MNRF provides an organizing conceptual model for mapping these interconnected dimensions and generating hypotheses regarding maternal and newborn healthcare resilience. Future research should prioritize underrepresented CESEE countries and use standardized, multicountry approaches to facilitate comparison across healthcare systems. Further studies should examine inequalities in access and quality of care, longer-term maternal and neonatal outcomes, and the effectiveness of healthcare adaptations.

## 5. Limitations

This review has several limitations. First, the available evidence was heterogeneous in study design, population, healthcare setting, data source, and reported outcomes, limiting direct comparisons across publications and countries. Most evidence was observational; therefore, changes in maternal, perinatal, and neonatal outcomes observed during the pandemic cannot be causally attributed to healthcare-system adaptations or disruptions. Second, the geographical distribution of the evidence was uneven, with eligible publications concentrated in a limited number of CESEE countries. Restriction to English-language publications may have disproportionately reduced the retrieval of evidence from countries where maternal and newborn healthcare research is more frequently disseminated in national languages or regional journals. In addition, the exclusion of reports for which full text could not be retrieved, limitations in database coverage, and the absence of a systematic gray-literature search may have further reduced the amount of regional evidence captured. Consequently, the observed geographical gaps reflect the evidence identified under the predefined eligibility and search criteria and should not be interpreted as demonstrating an absence of relevant research in those countries. The findings should therefore not be considered representative of CESEE healthcare systems as a whole. Third, several publications were derived from shared or partially overlapping datasets, particularly within the IMAgiNE EURO project and the Romanian population-based analyses, and should therefore not be interpreted as fully independent evidence. Some survey-based studies were also susceptible to self-selection and recall bias. Additionally, 12 reports sought for retrieval could not undergo full-text eligibility assessment because the full text was unavailable, including conference abstracts for which a corresponding full-text publication could not be identified or retrieved. Consequently, the possibility that potentially relevant evidence was missed cannot be excluded. Finally, the included publications covered different periods of the pandemic, during which epidemiological conditions, restrictions, and healthcare-service organization evolved substantially. Temporal distinctions reported by the primary publications were retained where available, but the heterogeneity of study periods and country-specific policy contexts precluded consistent classification into standardized pandemic phases and limited direct comparison over time. Although the MNRF domains were predefined and publications were independently mapped using explicit operational definitions, some interpretive subjectivity remains possible. Moreover, the MNRF has not undergone external validation or prospective testing and should therefore be considered an organizing conceptual model rather than a validated instrument for measuring healthcare-system resilience. Two included publications were co-authored by members of the review team. To minimize potential reviewer-related bias, their eligibility, extracted data, and MNRF domain assignment were independently rechecked during revision by a co-author who was not involved in either original study.

## 6. Conclusions

The COVID-19 pandemic exposed important vulnerabilities and prompted substantial adaptations in maternal and newborn healthcare delivery in the represented CESEE settings. Disruptions affected access and continuity of care, service organization, and several components of quality and family-centered care, while the available evidence did not consistently demonstrate widespread deterioration in maternal, perinatal, and neonatal outcomes. The MNRF provides a structured, hypothesis-generating conceptual model for organizing evidence across interconnected dimensions relevant to maternal and newborn healthcare resilience and for identifying priorities for preparedness and future research. Its applicability and external validity require prospective evaluation across different healthcare settings and public health emergencies.

## Figures and Tables

**Table 1:** Population–Concept–Context framework.

PCC Component	Description
Population	Pregnant women, postpartum women, newborns, healthcare professionals, and maternity and neonatal healthcare services
Concept	Maternal and newborn healthcare delivery, encompassing access to and continuity of care, organization and adaptation of maternity and neonatal services, clinical and family-centered care practices, and maternal, perinatal, and neonatal outcomes potentially associated with changes in healthcare delivery
Context	The predefined CESEE countries listed in Section 2.1 during the COVID-19 pandemic

**Table 2:** Characteristics of the included studies and their contribution to the MNRF.

Study	Country/ Region	Design	Population/ Sample	MNRF Domains	Primary Theme	Key Findings
1. Lazzerini et al., 2022 [21]	12 WHO European countries	Cross-sectional survey	21,027 women	A, H, Q	QMNC	Difficulty accessing antenatal care, companion restrictions, inadequate breastfeeding support, lower QMNC in several CESEE countries.
2. Drandić et al., 2022 [26]	Slovenia, Croatia, Serbia, Bosnia–Herzegovina	Cross-sectional survey	4817 women	A, H, Q	QMNC in former Yugoslav countries	Reduced access to antenatal care, high companion restrictions, inadequate breastfeeding support, perceived reduction in care quality.
3. Otelea et al., 2022 [27]	Romania	Cross-sectional survey	620 women	A, H, Q	Perinatal care quality	Deficiencies in breastfeeding, skin-to-skin, rooming-in, communication and decision-making, especially after cesarean birth.
4. Pumpure et al., 2022 [28]	Latvia	Cross-sectional survey	2079 women	A, H, Q	QMNC	Reduced perceived quality, inadequate breastfeeding support, limited involvement in choices, reported abuse; improvement from 2020 to 2021.
5. Kalita-Kurzyńska et al., 2024 [29]	Poland	Cross-sectional survey	636 pregnant women	A, H, Q	Maternity care experience	Partner restrictions, teleconsultations, cancelations, and reduced appointment duration.
6. Czuba et al., 2022 [30]	Poland	Nationwide cross-sectional	237,396 women	A, H	Prenatal screening continuity	Decreased prenatal screening program participation; fewer invasive diagnostic procedures.
7. Szczygioł et al., 2022 [31]	Poland	Prospective observational	83 mothers, 84 newborns	Q, O	Perinatal management	High skin-to-skin/rooming-in and breastfeeding rates despite SARS-CoV-2 infection.
8. Kanecki et al., 2024 [32]	Poland	Nationwide retrospective registry study	104.450 neonatal hospitalizations	H, O	Neonatal hospitalizations before and during the COVID-19 pandemic	Increased neonatal hospitalization rates increased during the pandemic. Hospitalizations related to maternal pregnancy complications, gestational diabetes, and placental disorders became more frequent, while prematurity and neonatal mortality remained largely unchanged.
9. Kalanj et al., 2025 [33]	Croatia	National comparative cross-sectional	National DRG data	A, H	Health system utilization	Marked decline in obstetric/gynecologic admissions during the pandemic, indicating substantial changes in service utilization.
10. Dagelić et al., 2023 [34]	Croatia	Retrospective cross-sectional study	691 miscarriages; 96 fetal/neonatal deaths	H, O	Adverse pregnancy outcomes	No significant differences were observed in miscarriage, stillbirth, or early neonatal death between the pre-pandemic and pandemic periods.
11. Galis et al., 2024 [15]	Romania	Population-based retrospective cohort	21,378 births	A, H, O	Perinatal outcomes	Stillbirth increased during pandemic; preterm birth modestly decreased.
12. Trif et al., 2025 [16]	Romania	Population-based retrospective cohort	52,021 births	A, H, O	Seasonality/outcomes	Preterm birth stable; stillbirth increased during strict lockdown.

Abbreviations: A = access and continuity of care; H = healthcare organization and system capacity; Q = quality and family-centered care; O = maternal, perinatal, and neonatal outcomes.

## Data Availability

No new data were created or analyzed in this study. Data sharing is not applicable to this article.

## Supplementary Materials

The following supporting information can be downloaded at https://www.mdpi.com/article/10.3390/medicina62101900/s1. Table S1. JBI critical appraisal of included publications. Table S2. Geographical distribution of the included evidence across the predefined CESEE countries. Supplementary File S1: Search strategy. Supplementary File S2: Preferred Reporting Items for Systematic reviews and Meta-Analyses extension for Scoping Reviews (PRISMA-ScR) Checklist.
