# Supplementary material for: Impacts of COVID-19 on Maternal and Newborn Healthcare Delivery in Central, Eastern and Southeastern Europe: A Scoping Review and Proposal for a Resilience Framework
Source: Medicina (Kaunas). 2026 Oct 1;62(10):1900. doi: 10.3390/medicina62101900 (PMC13707023; doi:10.3390/medicina62101900)
Supplement: Supplementary file 1 [file medicina-62-01900-s001.zip › Supplementary File S1.pdf]

Search strategy

DATABASE	SEARCH TERMS
**PUBMED**	("COVID-19" OR "SARS-CoV-2") AND (pregnancy OR maternal OR obstetric OR neonatal OR newborn) AND ("healthcare delivery" OR "quality of care" OR "antenatal care" OR telemedicine OR breastfeeding OR "rooming-in" OR "family-centered care") AND (Romania OR Poland OR Croatia OR Serbia OR Slovenia OR Slovakia OR Latvia OR Lithuania OR Estonia OR Hungary OR Czech Republic OR Bulgaria OR Bosnia and Herzegovina OR Moldova OR Montenegro OR Albania OR North Macedonia)
**SCOPUS**	TITLE-ABS-KEY(("COVID-19" OR "SARS-CoV-2" OR pandemic) AND (obstetric* OR matern* OR pregnan* OR newborn* OR neonatal* OR childbirth* OR delivery* OR NICU OR "prenatal care" OR "perinatal care") AND (healthcare OR "health care" OR telemedicine OR breastfeeding OR "rooming-in" OR "skin-to-skin" OR "family-centered care" OR "quality of care" OR communication OR "prenatal screening" OR "antenatal care" OR "health services accessibility" OR "delivery of health care") AND (Romania OR Poland OR Croatia OR Serbia OR Slovenia OR Slovakia OR Latvia OR Lithuania OR Estonia OR Hungary OR "Czech Republic" OR Bulgaria OR Bosnia OR Moldova OR Montenegro OR Albania OR "North Macedonia"))
**WEB OF SCIENCE**	TS=(("COVID-19" OR "SARS-CoV-2" OR pandemic) AND (obstetric* OR matern* OR pregnan* OR newborn* OR neonatal* OR childbirth* OR delivery* OR NICU OR "prenatal care" OR "perinatal care") AND (healthcare OR "health care" OR telemedicine OR breastfeeding OR "rooming-in" OR "skin-to-skin" OR "family-centered care" OR "quality of care" OR communication OR "prenatal screening" OR "antenatal care") AND (Romania OR Poland OR Croatia OR Serbia OR Slovenia OR Slovakia OR Latvia OR Lithuania OR Estonia OR Hungary OR "Czech Republic" OR Bulgaria OR Bosnia OR Moldova OR Montenegro OR Albania OR "North Macedonia"))

Google Scholar

Google Scholar was not treated as a primary database. Its role was to identify records not indexed in PubMed/MEDLINE, Scopus, or Web of Science, particularly from national and regional CESEE journals with incomplete indexing in those three sources. Because Google Scholar does not support reproducible Boolean syntax, bulk export, or stable result ordering, three grouped queries were run — grouping rather than 17 single-country queries, to keep the number of screened result pages manageable — collectively covering all 17 CESEE countries defined in Section 2.1:(COVID-19 OR pandemic) (maternal OR pregnancy OR newborn) (healthcare OR telemedicine) (Poland OR Slovakia OR Latvia OR Lithuania OR Estonia OR Hungary OR "Czech Republic")

(COVID-19 OR pandemic) (maternal OR pregnancy OR newborn) (healthcare OR telemedicine) (Romania OR Croatia OR Serbia OR Slovenia OR Bulgaria OR Bosnia)

Albania, Moldova, Montenegro, North Macedonia): (COVID-19 OR pandemic) (maternal OR pregnancy OR newborn) (healthcare OR telemedicine) (Albania OR Moldova OR Montenegro OR "North Macedonia")

For each query, results were sorted by relevance. Screening followed a pre-specified stopping rule: the first 200 results per query, or until two consecutive pages (20 results) yielded no new eligible record, whichever came first.
