# Supplementary material for: Impacts of COVID-19 on Maternal and Newborn Healthcare Delivery in Central, Eastern and Southeastern Europe: A Scoping Review and Proposal for a Resilience Framework
Source: Medicina (Kaunas). 2026 Oct 1;62(10):1900. doi: 10.3390/medicina62101900 (PMC13707023; doi:10.3390/medicina62101900)
Supplement: Supplementary file 1 [file medicina-62-01900-s001.zip › Supplementary Table S1.pdf]

Supplementary Table S1. JBI critical appraisal of included publications

Item-level judgments are reported as Y = Yes, N = No, U = Unclear, and N/A = Not applicable. Appraisal findings were used to characterize methodological strengths and limitations and were not used to exclude or formally weight publications.

S1A. Analytical cross-sectional studies

Publication	Q1	Q2	Q3	Q4	Q5	Q6	Q7	Q8	Key appraisal note
Lazzerini et al., 2022 [20]	Y	Y	Y	N/A	Y	Y	Y	Y	Validated WHO standards-based instrument and adjusted analyses; non-probability online recruitment limits representativeness.
Drandić et al., 2022 [25]	Y	Y	Y	N/A	Y	Y	Y	Y	Validated instrument and multivariable analysis; self-selection, higher education/primiparity and unequal country samples.
Otelea et al., 2022 [26]	Y	Y	Y	N/A	Y	Y	Y	Y	Validated instrument with confounder adjustment; voluntary online sample not representative of all Romanian mothers.
Pumpure et al., 2022 [27]	Y	Y	Y	N/A	Y	Y	Y	Y	Validated instrument and multivariable analysis; online recruitment and recall bias for 2020 births remain relevant.
Kalita-Kurzyńska et al., 2024 [28]	Y	Y	Y	N/A	U	N	U	Y	Clear eligibility and adequate sample size, but self-selected social-media recruitment, no reported questionnaire validation, and no confounder adjustment.
Czuba et al., 2022 [29]	Y	Y	Y	Y	Y	Y	Y	Y	Nationwide standardized administrative data; normalization by annual deliveries addressed declining birth numbers, but private-sector/NIPT-related residual confounding remains.
Kanecki et al., 2024 [31]	Y	Y	Y	Y	Y	N	Y	Y	Nationwide mandatory registry with standardized ICD-10 data; limited maternal covariates and no multivariable adjustment.
Kalanj et al., 2025 [32]	Y	Y	Y	Y	Y	N	Y	Y	Broad national DRG coverage and standardized coding; lacks granular patient-level confounders and adjusted analyses.
Dagelić et al., 2023 [33]	Y	Y	Y	Y	Y	N	Y	Y	Clearly defined hospital-based pre/post comparison using clinical/pathology records and ReCoDe classification; no multivariable confounder adjustment.

Cross-sectional checklist items: Q1 inclusion criteria clearly defined; Q2 subjects and setting described; Q3 exposure measured validly/reliably; Q4 objective standard criteria used for the condition; Q5 confounding factors identified; Q6 strategies to address confounding stated; Q7 outcomes measured validly/reliably; Q8 appropriate statistical analysis.

S1B. Cohort studies

Publication	Q1	Q2	Q3	Q4	Q5	Q6	Q7	Q8	Q9	Q10	Q11	Key appraisal note
Galiș et al., 2024 [14]	Y	Y	Y	Y	N	N/A	Y	N/A	Y	N/A	Y	Matched historical periods from the same county population and standardized outcome definitions; no multivariable adjustment for confounding. Partial Bihor overlap with Trif et al.
Trif et al., 2025 [15]	Y	Y	Y	Y	N	N/A	Y	N/A	Y	N/A	Y	Large two-county population cohort with complete key outcome data; several maternal/healthcare covariates unavailable and no multivariable confounder adjustment. Partial Bihor overlap with Galiș et al.

Cohort checklist items: Q1 groups similar/recruited from same population; Q2 exposure measured similarly; Q3 exposure measured validly/reliably; Q4 confounders identified; Q5 strategies to address confounding; Q6 participants free of outcome at exposure; Q7 outcomes measured validly/reliably; Q8 follow-up sufficient; Q9 follow-up complete; Q10 strategies for incomplete follow-up; Q11 appropriate statistical analysis. N/A was used where perinatal outcomes were ascertained at birth from complete registry cohorts rather than through participant-level longitudinal follow-up.

S1C. Case series

Publication	Q1	Q2	Q3	Q4	Q5	Q6	Q7	Q8	Q9	Q10	Key appraisal note
Szczygioł et al., 2022 [30]	Y	Y	Y	U	Y	Y	Y	Y	Y	Y	Clear RT-qPCR case definition and detailed clinical/perinatal reporting; all confirmed cases in the study period appear to have been included, but consecutive inclusion was not explicitly stated. Single-center descriptive series without a comparator.

Case-series checklist items: Q1 clear inclusion criteria; Q2 condition measured in a standard/reliable way; Q3 valid methods used to identify the condition; Q4 consecutive inclusion; Q5 complete inclusion; Q6 demographics clearly reported; Q7 clinical information clearly reported; Q8 outcomes/follow-up clearly reported; Q9 site/clinic demographic information clearly reported; Q10 appropriate statistical analysis.

Interpretation: no summary quality score or high/moderate/low category was assigned. The item-level appraisal should be interpreted together with the study-design and evidence-source information in Supplementary Table S1.5.
