# Supplementary material for: Impacts of COVID-19 on Maternal and Newborn Healthcare Delivery in Central, Eastern and Southeastern Europe: A Scoping Review and Proposal for a Resilience Framework
Source: Medicina (Kaunas). 2026 Oct 1;62(10):1900. doi: 10.3390/medicina62101900 (PMC13707023; doi:10.3390/medicina62101900)
Supplement: Supplementary file 1 [file medicina-62-01900-s001.zip › Supplementary Table S2.pdf]

Supplementary Table S2. Geographical distribution of the included evidence across the predefined CESEE countries

Country	Country-specific/subregional publications (n)	Reported participant/record base	Major evidence represented / evidence gap
Albania	0	—	No eligible country-specific or subregional publication identified.
Bosnia and Herzegovina	1	Drandić et al.: 4,817 women across Bosnia and Herzegovina, Croatia, Serbia, and Slovenia (combined sample)	IMAgiNE EURO evidence on access and quality/family-centered care; substantial restrictions in companionship and variation in communication, respectful care, breastfeeding support, and other QMNC indicators.
Bulgaria	0	—	No eligible country-specific or subregional publication identified.
Croatia	3	Drandić et al.: 4,817 women across four countries (combined); Kalanj et al.: national DRG data; Dagelić et al.: 691 miscarriages and 96 fetal/neonatal deaths	Evidence on quality/family-centered care, healthcare utilization, and pregnancy/perinatal outcomes. Hospital admissions changed during the pandemic; no significant pre-/pandemic difference in selected adverse fetal outcomes in the Croatian observational study.
Czech Republic	0	—	No eligible country-specific or subregional publication identified.
Estonia	0	—	No eligible country-specific or subregional publication identified.
Hungary	0	—	No eligible country-specific or subregional publication identified.
Latvia	1	Pumpure et al.: 2,079 women	IMAgiNE EURO evidence on QMNC, including breastfeeding support, involvement in choices, reported abuse, and improvement in several indicators from 2020 to 2021.
Lithuania	0	—	No eligible country-specific or subregional publication identified.
Moldova	0	—	No eligible country-specific or subregional publication identified.
Montenegro	0	—	No eligible country-specific or subregional publication identified.
North Macedonia	0	—	No eligible country-specific or subregional publication identified.
Poland	4	Kalita-Kurzyńska et al.: 636 pregnant women; Czuba et al.: 237,396 women; Szczygioł et al.: 83 mothers/84 newborns; Kanecki et al.: 104,450 neonatal hospitalizations	Evidence on maternity-care experience, teleconsultation and partner restrictions, prenatal screening continuity, management of SARS-CoV-2-positive mother–newborn dyads, and neonatal healthcare utilization/outcomes.
Romania	3	Otelea et al.: 620 women; Galiș et al.: 21,378 births; Trif et al.: 52,021 births	Evidence on QMNC and family-centered care, antenatal access, preterm birth and stillbirth. The Bihor County data in Galiș et al. partially overlap with the Bihor component of Trif et al.
Serbia	1	Drandić et al.: 4,817 women across Bosnia and Herzegovina, Croatia, Serbia, and Slovenia (combined sample)	IMAgiNE EURO evidence on access and quality/family-centered care, including companionship, communication, shared decision-making, breastfeeding support, privacy, and respectful care.
Slovakia	0	—	No eligible country-specific or subregional publication identified.
Slovenia	1	Drandić et al.: 4,817 women across Bosnia and Herzegovina, Croatia, Serbia, and Slovenia (combined sample)	IMAgiNE EURO evidence on access and quality/family-centered care, with several indicators differing from the other countries in the subregional analysis.

Notes:

1. CESEE = Central, Eastern and Southeastern Europe; QMNC = quality of maternal and newborn care; DRG = diagnosis-related group.
2. The multinational Lazzerini et al. IMAgiNE EURO publication (21,027 women across 12 WHO European Region countries) is not added to the country-specific publication counts above because it represents the overarching multicountry survey and overlaps with country-specific/subregional IMAgiNE EURO analyses retained in the review.
3. For Drandić et al., the 4,817 participants represent the combined sample from Slovenia, Croatia, Serbia, and Bosnia and Herzegovina; a country-specific participant count is therefore not inferred in this summary.
4. Participant/record figures are reported as presented in the included publications and should not be summed to estimate a unique regional sample. In particular, IMAgiNE EURO publications share an overarching survey dataset, and the Romanian population-based publications contain partially overlapping Bihor County data.
5. A value of 0 indicates that no eligible country-specific or subregional publication was identified within the predefined search strategy and eligibility criteria; it should not be interpreted as evidence that no relevant research was conducted in that country.
